# PG-MCTFormer: A Prior-Guided Multi-Scale Convolutional Transformer for Interpretable Motor Imagery EEG Classification

**DOI:** 10.3390/biomimetics11060377

**Published:** 2026-05-30

**Authors:** Jiahui Yuan, Rui Zhang, Yazhou Zhao, Weidong Zhou, Lan Tian, Guoyang Liu

**Affiliations:** 1 School of Integrated Circuits, Shandong University, Jinan 250199, China; 2 Department of Biomedical Engineering, New York University, New York, NY 10012, USA; 3 Shenzhen Research Institute of Shandong University, Shenzhen 518000, China; 4 Key Laboratory of Social Computing and Cognitive Intelligence, Dalian University of Technology, Ministry of Education, Dalian 116024, China

**Keywords:** motor imagery electroencephalography (MI-EEG), brain–computer interface (BCI), deep learning, transformer

## Abstract

Motor imagery brain–computer interfaces (MI-BCIs) have important applications in neurorehabilitation, assistive communication, and non-muscular human–machine interaction. From a bionic neural-interfacing perspective, MI-BCI decoding provides a computational bridge between biological motor intention and external machine control. However, reliable motor imagery electroencephalography (MI-EEG) classification remains challenging due to the highly non-stationary features of MI-EEG and limited interpretability. In this work, we propose PG-MCTFormer, a prior-guided multi-scale convolutional Transformer for MI-EEG classification that integrates rhythm-aware temporal filtering, dual-scale spatial modeling, and contextual decoding within a unified architecture. We evaluated the model on the publicly available BCI Competition IV 2a dataset, achieving 85.08% average accuracy and a Cohen’s kappa of 0.80, with significant performance improvement over the traditional methods. Comprehensive multi-view interpretability analyses in the frequency, temporal, and spatial domains further show that the learned filters remain aligned with canonical MI-related bands, discriminative evidence concentrates in the middle-to-late imagery interval, and the spatial prior is refined into subject-adaptive sensorimotor topographic patterns. These results indicate that explicit neurophysiological priors can improve both the robustness and the interpretability of MI-EEG decoders for biomimetic neural-interface applications.

## 1. Introduction

Motor imagery (MI)-based brain–computer interfaces (BCIs) aim to decode neural activity associated with imagined movements into external control commands and play an important role in neurorehabilitation, assistive communication, and non-muscular human–machine interaction [1,2,3]. However, in scalp electroencephalography (EEG), reliable classification remains challenging due to the weak and non-stationary characteristics of discriminative patterns. This difficulty is further compounded by volume conduction and pronounced inter-subject variability [4,5,6].

From the perspective of bionic neural interfacing and rehabilitation, MI-BCI provides a representative paradigm in which endogenous sensorimotor activity is translated into machine-readable control information. EEG-based BCI systems convert task-related brain oscillations into actionable commands, while MI-related cortical activity provides a non-muscular pathway for decoding internal motor intention [7]. In rehabilitation applications, such decoded motor intentions can further be coupled with external assistive devices, including soft robotic systems, to support motor function recovery [8]. Therefore, improving the reliability and interpretability of MI-EEG decoding is relevant to bionic neural-interface applications, because it strengthens the neural decoding module that links biological motor intention with external machine control.

Traditional machine learning methods have been used for MI-EEG classification, typically following staged pipelines in which discriminative features are manually designed and then classified by conventional decision rules [9,10]. In spatial filtering, common spatial pattern (CSPs) is a widely used classic approach, which learns spatial filters that maximize variance differences between classes and thereby improve the separability of motor imagery tasks [11]. Building on this principle, robust, sparse, and transfer-regularized variants of CSP were later introduced to improve adaptation under noise, limited training data, and cross-subject variability [12,13,14]. For frequency modeling, filter-bank common spatial pattern (FBCSP) explicitly exploits MI-related spectral structure by decomposing EEG into multiple sub-bands and extracting spatial features within each band, and related sub-band and filter-bank extensions follow the same general strategy of combining band decomposition with spatial filtering [15]. In addition to spatial filtering and spectral modeling, some studies also introduced temporal structure into feature extraction. For example, local temporal common spatial pattern (LTCSP) emphasized discriminative spatial features within local temporal windows, whereas common spatio-time-frequency pattern learning jointly models spatial weights, frequency characteristics, and temporal window selection [16,17]. Meanwhile, another work represented EEG trials through covariance geometry by using Riemannian geometry and tangent-space mapping, and multiband tangent-space methods further combine sub-band decomposition with geometric representations [18]. After feature extraction, the resulting feature vectors or covariance descriptors were then classified using linear discriminant analysis (LDA), support vector machines (SVMs), or minimum-distance-based rules [19,20]. Nevertheless, these methods still depend heavily on manually specified band decomposition, feature extraction, and classifier design, which limits their ability to model the complex temporal, spectral, and spatial coupling structure of MI-EEG within a unified trainable framework.

Deep learning has driven a shift in MI-EEG from handcrafted feature engineering toward end-to-end representation learning [21,22]. Early studies showed that convolutional neural networks (CNNs) can learn task-relevant spatiotemporal representations directly from raw or minimally processed EEG, thereby reducing reliance on manually designed features [23,24]. Lawhern et al. [25] proposed EEGNet, which employs depthwise and separable convolutions to form a compact architecture for joint temporal and spatial modeling with a low parameter count. Subsequent studies further improved MI-EEG representation learning through multi-branch convolution, temporal modeling, and attention mechanisms. For example, Zhao et al. [26] introduced a multi-branch 3D convolutional network to extract multiscale spatiotemporal features through parallel branches. Ingolfsson et al. [27] incorporated temporal convolutional networks (TCNs) into MI-EEG decoding. Recently, Altaheri et al. [28] introduced an attention-based temporal convolutional architecture to enhance the modeling of informative temporal segments. To better capture global contextual dependencies, convolution–Transformer hybrid architectures have emerged as an important direction for MI-EEG decoding. Song et al. [29] proposed EEG Conformer, which combines convolutional embedding with self-attention to jointly model local features and global correlations in EEG. Following this line, Zhao et al. [30] developed CTNet, in which an EEGNet-like convolutional feature extractor is combined with a Transformer encoder to model global dependencies of high-level EEG representations. Altaheri et al. [31] further proposed TCFormer, which strengthens temporal-context modeling by introducing a multi-kernel CNN front-end, a Transformer encoder, and a TCN head. While this design improves the modeling of local and global temporal dependencies, it also indicates the increasing complexity of recent Transformer-based MI-EEG decoders. To improve the compactness of Transformer-based MI-EEG decoders, Keutayeva et al. [32] proposed EEGCCT, a compact convolutional Transformer designed for subject-independent MI-EEG decoding. Overall, these methods have substantially enhanced the end-to-end modeling capability of MI-EEG decoders. However, in many existing models, temporal filtering and spatial modules lack interpretability. Accordingly, such high-capacity models may be more vulnerable to overfitting and spurious correlations unless physiologically grounded constraints are incorporated into the learning process.

Recent studies have started to incorporate explicit time-frequency and spatial structure into MI-EEG modeling. For instance, Liu et al. [33] proposed a multiscale time-frequency method that identifies discriminative time-frequency segments through multiscale segmentation and wrapper-based selection, while Zhang et al. [34] combined self-attention-based CNNs with time-frequency common spatial pattern analysis to integrate time-frequency and spatial information more explicitly. For spatial modeling, the Parallel-Hierarchical Neural Network (PHNN) learns MI representations from brain-region-level features and progressively integrates them through multi-level fusion [35]. Beyond regional decomposition, graph-based models further characterize structured relationships among EEG channels. For example, Wang et al. [36] proposed NexusNet, which introduces lightweight graph modeling with multi-node routing and Nexus Fusion to capture higher-order channel relationships in MI-BCI decoding. Along this direction, brain-topography graph embedded CNNs explicitly construct topology-aware channel representations through graph embedding, while topology-aware multiscale fusion networks further incorporate spectral–topological information into EEG representation learning [37,38]. Integrated spatial-frequency-time frameworks have also been explored. For example, Liu et al. [39] proposed a fine-grained spatial-frequency-time framework that jointly performs time-frequency segmentation and spatial segmentation to obtain more discriminative segments. These studies highlight the value of explicit time-frequency and spatial modeling for MI decoding. Nevertheless, explicit rhythm-related temporal priors, flexible region-level spatial priors, and modern contextual classification are seldom coupled within a single architecture.

To address these limitations, we propose PG-MCTFormer, a prior-guided multi-scale convolutional Transformer for MI-EEG classification. By integrating the Multi-Scale Sinc Temporal Filterbank (MS-STF) and the Gated Dual-Branch Spatial Clustering module (GDB-SC) into the Rhythm-Preserved Spatiotemporal Modeler (RP-STM), the proposed model combines rhythm-aware temporal filtering, dual-scale spatial organization, and contextual classification within a unified end-to-end architecture. The main contributions of this study are summarized as follows:

- We propose the PG-MCTFormer, incorporating comprehensive temporal and frequency prior information of the MI-EEG to improve the model performance.
- We introduce a Multi-Scale Sinc Temporal Filterbank (MS-STF) module, which parameterizes temporal convolutions as Sinc-based finite-impulse-response (FIR) filters aligned with the theta, mu, and beta rhythms.
- We develop a Gated Dual-Branch Spatial Clustering module (GDB-SC) to jointly capture electrode-level and region-level spatial features, enabling multi-scale spatial representation learning.
- We demonstrate competitive MI-EEG classification performance on the widely used BCI Competition IV 2a dataset and provide comprehensive multi-view interpretability analyses in the frequency, temporal, and spatial domains.

## 2. Dataset

We evaluated the proposed model on the BCI Competition IV 2a dataset, a public four-class MI benchmark comprising left-hand, right-hand, both-feet, and tongue tasks [5,6]. The dataset contains recordings from nine subjects acquired on two different days. Each subject completed two sessions, each consisting of six runs of 48 trials, yielding 288 trials per session. EEG signals were recorded at 250 Hz from 22 Ag/AgCl electrodes arranged, together with three monopolar EOG channels. In this study, only the 22 EEG channels were used.

**Figure 1:**
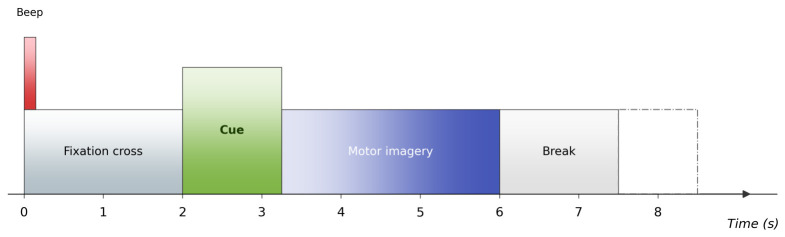
Experimental paradigm of BCI Competition IV 2a dataset.

Following the official session-wise evaluation protocol, the first session was used for training, and the second session was used for testing. Figure 1 illustrates the cue-based trial paradigm. At t=0 s, a fixation cross and warning tone were presented; at t=2 s, a visual cue indicated one of the four MI classes; and the trial ended at t=6 s. Following common practice in MI-EEG classification, we extracted the 4 s interval from cue onset (2 s) to trial end (6 s) for analysis. A minimal preprocessing pipeline was adopted to preserve the original spatiotemporal structure of the EEG data. Specifically, per-channel z-score standardization was applied using statistics computed from the training session only and then applied consistently to the test session.

## 3. Methodology

### 3.1. Architecture Overview

**Figure 2:**
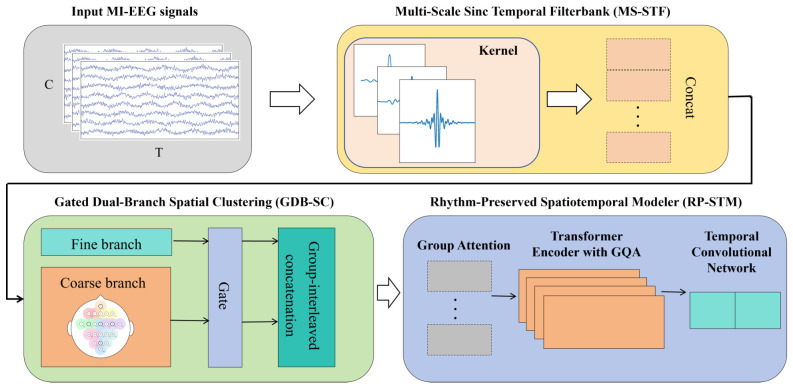
Overall architecture of PG-MCTFormer. The model integrates a prior-guided temporal frontend (MS-STF), a topology-guided spatial module (GDB-SC), and a rhythm-preserved spatiotemporal decoder (RP-STM).

Figure 2 shows the overall architecture of PG-MCTFormer. Given an input trial $X\in R^{B\times C\times T}$, where *B* is the batch size, C=22 is the number of EEG channels, and T=1000 is the number of time samples, the network proceeds through three stages. First, the Multi-Scale Sinc Temporal Filterbank (MS-STF) decomposes raw EEG into theta-, mu-, and beta-based temporal branches. Second, the Gated Dual-Branch Spatial Clustering module (GDB-SC) transforms the concatenated branch features into dual-scale spatial representations by combining electrode-level filtering with prior-guided region-level clustering. Third, the Rhythm-Preserved Spatiotemporal Modeler (RP-STM) preserves the rhythm-group structure while modeling local dynamics and global context through residual group attention, a grouped-query Transformer encoder, and a grouped TCN classification head.

The detailed mathematical formulations and configurations of these three core modules are elaborated in the following subsections.

### 3.2. The Multi-Scale Sinc Temporal Filterbank

Motor imagery EEG is commonly associated with modulations of sensorimotor rhythms, particularly in the mu and beta bands [40,41]. However, in scalp EEG, these canonical rhythm patterns are weak, spatially mixed, and subject-variable, limiting the effectiveness of fixed band definitions for robust temporal modeling. A suitable temporal frontend should therefore incorporate physiological spectral priors while retaining limited flexibility for subject-specific refinement. In this study, the MS-STF employs three parallel Sinc-based temporal branches corresponding to the theta, mu, and beta priors. Instead of learning arbitrary FIR filter coefficients, each branch learns only limited adjustments of the band boundaries, so that the resulting filters remain anchored to the target rhythms while still allowing moderate subject-specific adaptation.

**Figure 3:**
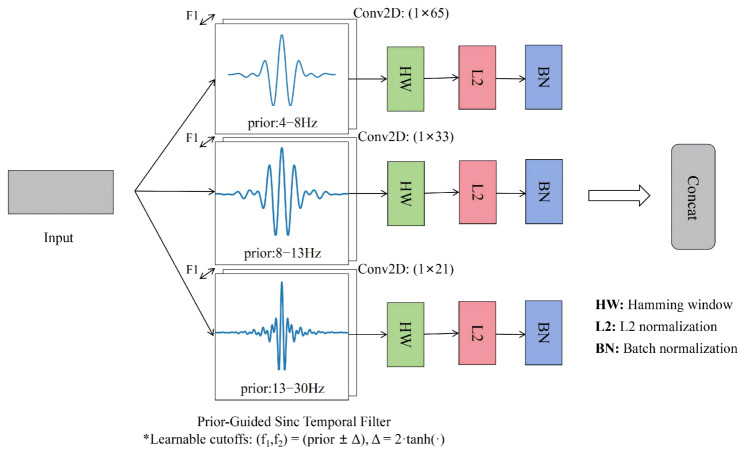
MS-STF schematic with theta/mu/beta kernel lengths of 65/33/21.

As illustrated in Figure 3, the input trial is first rearranged from $R^{B\times C\times T}$ to $R^{B\times 1\times C\times T}$ to perform temporal convolution while preserving the electrode dimension. The three branches are aligned with the theta [4–8 Hz], mu [8–13 Hz], and beta [13–30 Hz] priors and are assigned kernel lengths of 65, 33, and 21 samples, respectively. At the sampling rate of 250 Hz, these branches correspond to temporal supports of 260 ms, 132 ms, and 84 ms. In the reported configuration, each branch produces $F_{1}=32$ temporal feature maps.

Let g∈{θ,μ,β} denote the rhythm branch and let $m\in \{1,\ldots ,F_{1}\}$ denote the filter index. For branch *g*, let $\left[f_{l,0}^{\left(g\right)},f_{h,0}^{\left(g\right)}\right]$ denote the prior band and let $b_{0}^{\left(g\right)}=f_{h,0}^{\left(g\right)}-f_{l,0}^{\left(g\right)}$ be the corresponding prior bandwidth. The key design of MS-STF is that each filter learns a bounded low-cutoff offset and a bounded bandwidth offset rather than a fully free spectral profile:

(1) $$\Delta_{l,m}^{\left(g\right)}=\delta_{\max}\tanh\;\left(\rho_{l,m}^{\left(g\right)}\right),\;\Delta_{b,m}^{\left(g\right)}=\delta_{\max}\tanh\;\left(\rho_{b,m}^{\left(g\right)}\right),$$

where $\delta_{\max}=2$ Hz sets the maximum spectral adjustment. The resulting effective low cutoff, bandwidth, and high cutoff are

(2) $$f_{l,m}^{\left(g\right)}=\max\;\left(f_{\min},\;f_{l,0}^{\left(g\right)}+\Delta_{l,m}^{\left(g\right)}\right),\;b_{m}^{\left(g\right)}=\max\;\left(b_{\min},\;b_{0}^{\left(g\right)}+\Delta_{b,m}^{\left(g\right)}\right),$$

(3) $$f_{h,m}^{\left(g\right)}=\min\;\left(f_{\mathrm{Nyq}},\;f_{l,m}^{\left(g\right)}+b_{m}^{\left(g\right)}\right),$$

where $f_{\min}=1$ Hz and $b_{\min}=2$ Hz prevent degenerate bands, and $f_{\mathrm{Nyq}}=f_{s}/2$ with $f_{s}=250$ Hz is the Nyquist frequency. Using normalized frequencies $\bar{f}=f/f_{s}$, the corresponding windowed Sinc band-pass kernel is

(4) $$h_{m}^{\left(g\right)}\left[n\right]\propto w\left[n\right]\left(2{\bar{f}}_{h,m}^{\left(g\right)}\;\mathrm{sinc}\;\left(2{\bar{f}}_{h,m}^{\left(g\right)}n\right)-2{\bar{f}}_{l,m}^{\left(g\right)}\;\mathrm{sinc}\;\left(2{\bar{f}}_{l,m}^{\left(g\right)}n\right)\right),$$

where w[n] is a Hamming window. The kernel is $l_{2}$-normalized before convolution. In this way, the waveform is generated analytically, and only the band boundaries are adapted during training. This Sinc-based FIR parameterization substantially reduces the degrees of freedom of the temporal frontend relative to unconstrained filters.

Let $H^{\left(\theta \right)}$, $H^{\left(\mu \right)}$, and $H^{\left(\beta \right)}$ denote the outputs of the three temporal branches. These branch-wise outputs are concatenated as

(5) $$H_{\mathrm{temp}}=\mathrm{Cat}\left[H^{\left(\theta \right)},H^{\left(\mu \right)},H^{\left(\beta \right)}\right]\in R^{B\times (3F_{1})\times C\times T}.$$

Because temporal branch gating is disabled in the reported configuration, $H_{\mathrm{temp}}$ is passed directly to the spatial module. Figure 3 summarizes this process, showing the three prior-aligned temporal branches and their asymmetric kernel lengths.

### 3.3. The Gated Dual-Branch Spatial Clustering Module

Compared with the explicit temporal decomposition used in many MI-EEG decoders, spatial organization is still often handled in a more implicit manner. Existing deep learning models commonly rely either on global channel mixing across all electrodes or on a single spatial resolution, which makes it difficult to represent both localized sensorimotor patterns and broader network-level organization within the same spatial module [42,43,44]. The GDB-SC module is introduced to address this limitation by performing dual-scale spatial modeling on the temporal feature tensor $H_{\mathrm{temp}}\in R^{B\times GF_{1}\times C\times T}$, where G=3 denotes the number of rhythm groups. Specifically, GDB-SC decomposes spatial modeling into a fine electrode-level pathway and a coarse region-level pathway, and then fuses the two views through learnable group-wise branch weights.

The fine pathway is designed to preserve channel-level discriminative detail. It applies depthwise spatial filtering directly over the electrode dimension,

(6) $$Y_{\mathrm{fine}}={\mathrm{DWConv}}_{C\times 1}\left(H_{\mathrm{temp}}\right)\in R^{B\times (GF_{1}D)\times 1\times T},$$

where *D* denotes the spatial depth multiplier. Because the convolution kernel spans the full channel axis, this pathway learns spatial correlations directly from electrode-level activity and remains sensitive to localized MI patterns.

**Figure 4:**
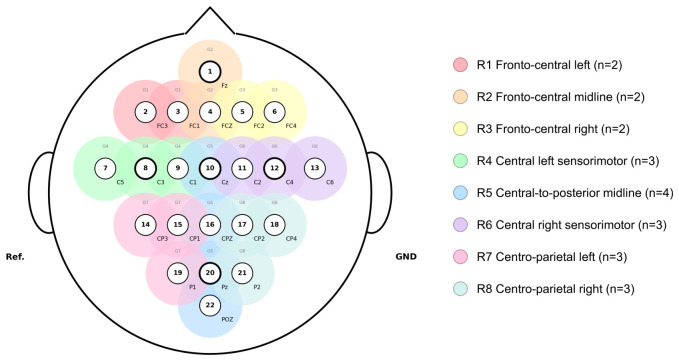
Eight-region motor prior on the 22-channel EEG electrode layout. R1–R8 denote the predefined sensor-level regions, and *n* indicates the number of EEG channels in each region. Ref. and GND mark the reference and ground positions, respectively.

The coarse pathway is designed to incorporate a physiologically motivated regional organization into the electrode-level representation. Specifically, following the 22-channel montage of BCI Competition IV 2a, the electrodes are grouped into eight motor-related sensor-level regions to initialize the electrode-to-region soft assignment matrix (W), as shown in Figure 4. This grouping separates left-lateralized, midline, and right-lateralized electrodes to preserve the lateralized sensorimotor organization commonly observed around C3 and C4 during hand motor imagery [45]. It further distinguishes fronto-central, central, and centro-parietal electrodes to retain the anterior–posterior organization of MI-related scalp activity, which is compatible with evidence that motor imagery involves distributed fronto-parietal components in addition to central sensorimotor rhythm modulation [46,47].

The learnable assignment matrix W further relaxes the predefined grouping template into an adaptive soft sensor-to-region mapping. A fixed hard partition may be overly restrictive for EEG spatial modeling, since scalp-level recordings exhibit spatial mixing and reference-dependent topographies, and the discriminative MI patterns may vary across subjects. This design enables the coarse pathway to learn region-level MI-related spatial representations while preserving flexibility for subject-specific spatial patterns.

Let $L_{\mathrm{learn}}\in R^{R\times C}$ denote the learnable assignment logits, let $L_{\mathrm{rand}}\in R^{R\times C}$ denote a randomly initialized logit matrix of the same shape, let $W_{0}\in R^{R\times C}$ denote the row-stochastic regional prior over the electrode layout, let ε denote a small numerical constant, and let R=8 for the reported eight-region configuration. In the reported setting, this prior is used for initialization rather than as a hard forward constraint:

(7) $$L_{\mathrm{learn}}^{\left(0\right)}=\left(1-a\right)L_{\mathrm{rand}}+a\;\frac{\log(W_{0}+\varepsilon )}{\tau_{\mathrm{init}}}$$

where a=0.3 controls the prior mixing ratio and $\tau_{\mathrm{init}}=2.0$ smooths the initialization. The learnable logits $L_{\mathrm{learn}}$ are initialized by $L_{\mathrm{learn}}^{\left(0\right)}$. During training and inference, the actual soft electrode-to-region assignment matrix is obtained by

(8) $$W=\mathrm{softmax}\;\left(\frac{L_{\mathrm{learn}}}{\tau_{c}}\right),$$

where the softmax is applied row-wise over electrodes and $\tau_{c}=1.2$ controls the sharpness of the assignments. In contrast to a hard region partition, this soft projection allows each region to retain partial contributions from multiple electrodes. In the reported configuration, the same W is shared across the three rhythm groups. The region-projected feature tensor is then computed as

(9) $${\left(H_{\mathrm{reg}}\right)}_{b,f,r,t}=\sum_{c=1}^{C}W_{r,c}{\left(H_{\mathrm{temp}}\right)}_{b,f,c,t},$$

which aggregates electrode features into region-level responses. A second depthwise spatial filtering stage is subsequently applied over the region axis,

(10) $$Y_{\mathrm{coarse}}={\mathrm{DWConv}}_{R\times 1}\left(H_{\mathrm{reg}}\right)\in R^{B\times (GF_{1}D)\times 1\times T}.$$

This coarse pathway captures broader sensorimotor network-level patterns that are difficult to represent by electrode-level filtering alone.

The outputs of the two pathways are fused by a learnable rather than a fixed gating structure. This design provides a compact and interpretable group-level branch weighting, where the fine/coarse preference is optimized during training but remains shared across trials within each temporal group. For temporal group *g*, let $l_{g}\in R^{2}$ denote the learnable fine/coarse gate logits and define

(11) $$\left[\alpha_{g},\beta_{g}\right]=\mathrm{softmax}\left(l_{g}\right),$$

where $\alpha_{g}$ and $\beta_{g}$ denote the learned contributions of the fine and coarse branches, respectively. These weights are broadcast to all $F_{1}D$ channels belonging to group *g*:

(12) $${\tilde{Y}}_{\mathrm{fine}}^{\left(g\right)}=\alpha_{g}Y_{\mathrm{fine}}^{\left(g\right)},\;{\tilde{Y}}_{\mathrm{coarse}}^{\left(g\right)}=\beta_{g}Y_{\mathrm{coarse}}^{\left(g\right)}.$$

Instead of using ordinary concatenation, the fused output is organized by group-aligned interleaving,

(13) $$Y_{\mathrm{out}}=\mathrm{Interleave}\left[{\tilde{Y}}_{\mathrm{fine}}^{\left(1\right)},{\tilde{Y}}_{\mathrm{coarse}}^{\left(1\right)},\ldots ,{\tilde{Y}}_{\mathrm{fine}}^{\left(G\right)},{\tilde{Y}}_{\mathrm{coarse}}^{\left(G\right)}\right],$$

so that fine- and coarse-scale spatial features belonging to the same rhythm remain aligned within the same computational group. In this way, GDB-SC preserves localized electrode information, incorporates region-level anatomical structure, and provides a spatial representation that is better matched to the grouped decoder used in the subsequent stage.

### 3.4. The Rhythm-Preserved Spatiotemporal Modeler

After the frontend, the feature has been organized into explicit rhythm-specific groups. The RP-STM operates on this grouped representation to model both local temporal evolution and global contextual dependencies. Let $d_{\mathrm{group}}$ denote the per-group channel width and let $d_{\mathrm{model}}=G\;d_{\mathrm{group}}$ denote the total grouped channel width after spatial fusion. The decoder first applies temporal pooling and dropout, followed by a grouped 1×1 reduction and a grouped temporal convolution:

(14) $$U_{1}=\mathrm{BN}\left({\mathrm{GConv}}_{1\times 1}\left(\mathrm{Drop}\left({\mathrm{Pool}}_{1}\left(Y_{\mathrm{out}}\right)\right)\right)\right),$$

(15) $$U_{2}=\phi \left(\mathrm{BN}({\mathrm{GConv}}_{1\times 16}\left(U_{1}\right))\right),$$

where ϕ(·) denotes the ELU activation. A residual group-attention block, denoted by $A_{\mathrm{grp}}\left(\cdot \right)$, is then applied,

(16) $${\hat{U}}_{2}=U_{2}+A_{\mathrm{grp}}\left(U_{2}\right),$$

followed by a second average-pooling stage and dropout to produce local grouped features $F_{\mathrm{loc}}\in R^{B\times d_{\mathrm{model}}\times T^{\prime }}$; in the reported configuration, $T^{\prime }=17$.

Before Transformer modeling, the local grouped feature map is projected by an ungrouped 1×1 convolution and rearranged into tokens, allowing controlled cross-group interaction before global contextual modeling:

(17) $$Z_{0}=\mathrm{Rearrange}\left(\psi \left(\mathrm{BN}({\mathrm{Conv}}_{1\times 1}\left(F_{\mathrm{loc}}\right))\right)\right)\in R^{B\times T^{\prime }\times d_{\mathrm{model}}},$$

where ψ(·) denotes the SiLU activation. Let *L* denote the number of Transformer blocks and let l=0,…,L−1 index the blocks. The token sequence is then processed by a stack of Transformer blocks with grouped-query self-attention (GQA) [48], shared key/value groups, and rotary positional embeddings (RoPE) [49]. In abstract form, each block performs

(18) $$Z_{l+\frac{1}{2}}=Z_{l}+\mathrm{GQA}\left(\mathrm{LN}(Z_{l})\right),$$

(19) $$Z_{l+1}=Z_{l+\frac{1}{2}}+\mathrm{MLP}(\mathrm{LN}(Z_{l+\frac{1}{2}})).$$

In the reported configuration, four query heads share two key/value groups, enabling efficient contextual modeling while retaining the grouped organization established by the prior-guided frontend.

The Transformer output is then projected to an additional grouped feature representation by a second 1×1 convolution,

(20) $$F_{\mathrm{tr}}=\psi \left(\mathrm{BN}({\mathrm{Conv}}_{1\times 1}\left(Z_{L}^{⊤}\right))\right)\in R^{B\times d_{\mathrm{group}}\times T^{\prime }},$$

and concatenated with the retained local grouped representation:

(21) $$F=\mathrm{Cat}\left[F_{\mathrm{loc}},F_{\mathrm{tr}}\right]\in R^{B\times \left(\left(G+1\right)d_{\mathrm{group}}\right)\times T^{\prime }}.$$

**Figure 5:**
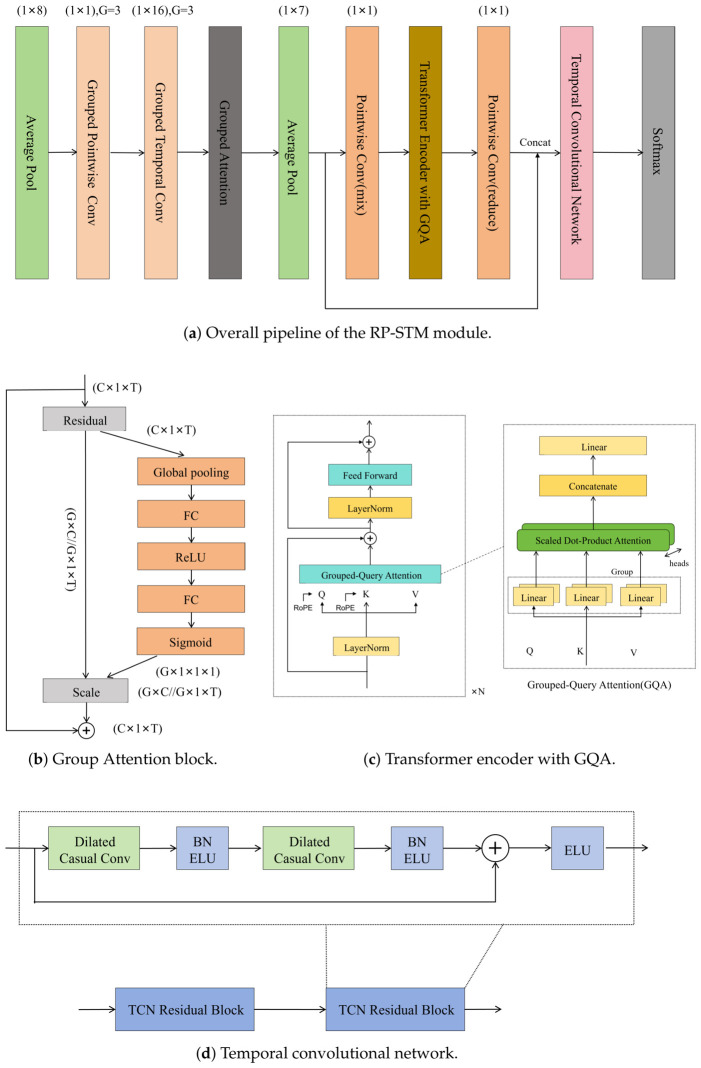
Detailed views of the RP-STM module. In addition to grouped attention and Transformer token mixing, the final decoder explicitly includes a grouped TCN head for temporal aggregation and classification.

Finally, the fused sequence is decoded by a grouped temporal convolutional network (TCN) head. Each residual TCN block contains two grouped causal convolutions with batch normalization, ELU activation, and dropout, and the two blocks use dilation factors of 1 and 2 [50]. Let $Q\in R^{B\times \left(\left(G+1\right)d_{\mathrm{group}}\right)\times T^{\prime }}$ denote the TCN output. The decoder then retains the final time step and applies a grouped 1×1 class projection; the resulting logits are averaged across groups to obtain the final prediction. Figure 5 summarizes the RP-STM decoder and its main components, including the overall pipeline, the residual group-attention block, the grouped-query Transformer encoder, and the grouped TCN head.

### 3.5. Data Augmentation

To mitigate overfitting under limited training data, a segmentation-and-reconstruction (S&R) augmentation strategy was applied to the training session only [51]. Each motor imagery trial $X_{i}\in R^{C\times T}$ was divided into $N_{s}$ non-overlapping temporal segments of equal length ($N_{s}=8$). Additional synthetic trials were then generated by randomly concatenating segments drawn from trials of the same class while preserving their original temporal order. This class-consistent reconstruction increases intra-class variability without altering label identity and without introducing cross-session information leakage. In our experiments, the number of augmented trials was set equal to the number of original training trials, so that the effective training set size was doubled.

### 3.6. Model Training and Testing

The network was trained by minimizing the cross-entropy loss. Consistent with the training configuration of TCFormer [31], the model was trained for 1000 epochs using the Adam optimizer with a learning rate of $0.9\times 10^{-3}$. The training objective is given by

(22) $$L_{\mathrm{CE}}=-\frac{1}{M}\sum_{i=1}^{M}\sum_{j=1}^{N}y_{ij}\log\left({\hat{y}}_{ij}\right),$$

where *M* is the batch size, *N* is the number of classes, $y_{ij}$ is the ground-truth indicator, and ${\hat{y}}_{ij}$ is the predicted posterior probability. The detailed training and testing procedure of PG-MCTFormer is summarized in Algorithm 1.
**Algorithm 1** Training and testing procedure of PG-MCTFormer**Require:**    Training session $D_{tr}$ and test session $D_{te}$    Motor-region prior matrix $W_{0}$    Rhythm-band priors **Ensure:**    Trained model M    Predicted labels for $D_{te}$  1: Standardize the training and test trials using statistics from $D_{tr}$;  2: Initialize the Sinc filters with rhythm-band priors;  3: Initialize the spatial logits using the motor-region prior matrix by Equation (7);  4: Initialize the remaining network parameters and the Adam optimizer;  5: **for** each training epoch **do**  6:    **for** each mini-batch from $D_{tr}$ **do**  7:     Apply same-class S&R augmentation to the current mini-batch;  8:     Extract rhythm-aware temporal features using MS-STF;  9:     Generate the soft electrode-to-region assignment matrix W; 10:     Extract topology-guided spatial features using GDB-SC; 11:     Decode the spatial features and obtain classification logits using RP-STM; 12:     Calculate the cross-entropy loss using Equation (22); 13:     Update the model parameters using Adam; 14:    **end for** 15: **end for** 16: Decode the test trials using the trained model M; 17: **return** M and the predicted test labels.


## 4. Results

**Figure 6:**
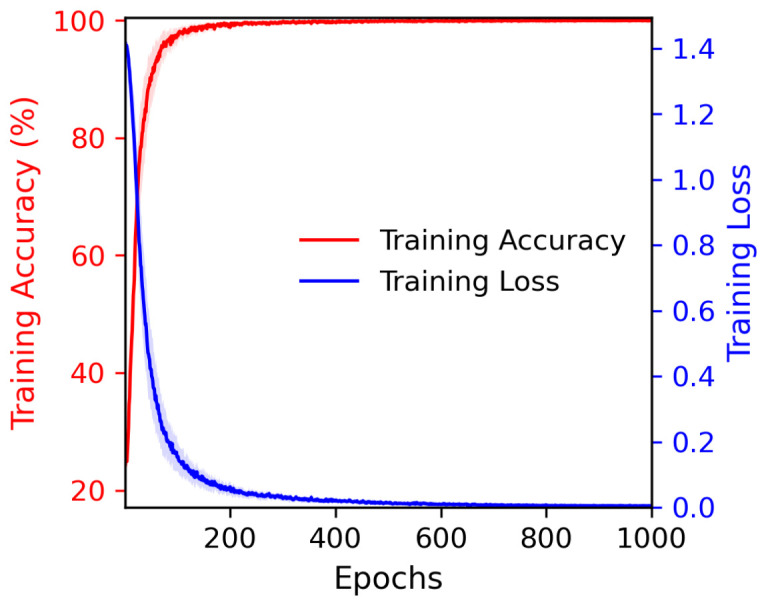
Training dynamics of the proposed model over 1000 epochs. The red curve denotes the training accuracy, and the blue curve denotes the training loss.

To evaluate training convergence, the average training accuracy and training loss were monitored. As shown in Figure 6, the proposed model reaches over 90% training accuracy within the first 50 epochs and gradually stabilizes after approximately 150–200 epochs, while the training loss drops rapidly from about 1.4 to below 0.2 and then decreases more slowly, indicating fast convergence and stable optimization.

Table 1 reports the classification performance comparison of representative baselines together with PG-MCTFormer on the BCI Competition IV 2a dataset under the official session-wise protocol. To ensure a fair and robust comparison, all models were reproduced using a unified experimental protocol. Specifically, the BCI Competition IV 2a data were processed with channel-wise z-score normalization and the same S&R augmentation strategy, and all models were trained using Adam optimization with a learning rate of $0.9\times 10^{-3}$ for 1000 epochs. The reported results were averaged over three independent random runs to reduce the influence of random initialization. The reproduced NexusNet result is lower than the 78.78% accuracy reported in the original study, because the original NexusNet setting used a dedicated filtering-and-standardization pipeline, especially 2–40 Hz FIR bandpass filtering followed by exponential moving standardization, together with 2000 training epochs, a learning rate of 0.005, and early stopping. The proposed model achieved the highest average accuracy of 85.08% and a Cohen’s κ of 0.80 on the BCI Competition IV 2a dataset.

To further evaluate the reliability of the observed improvements, paired two-sided *t*-tests were performed on the subject-wise accuracies between PG-MCTFormer and each baseline, followed by Benjamini–Hochberg false discovery rate (BH-FDR) correction for multiple comparisons. Before applying the paired *t*-tests, Shapiro–Wilk (SW) tests were conducted on the paired subject-wise accuracy differences, and no evidence against normality was observed for any comparison (p>0.05, SW test). Compared with EEGNet and NexusNet, PG-MCTFormer showed large performance margins, improving the average accuracy by 12.25% (p<0.001, 95% confidence interval: [8.83, 15.68], $d_{z}=2.752$, BH-FDR-corrected) and 16.65% (p=0.001, 95% confidence interval: [10.30, 23.01], $d_{z}=2.014$, BH-FDR-corrected), respectively. For convolution–Transformer hybrid baselines, PG-MCTFormer outperformed EEG Conformer by 9.71% (p=0.002, 95% confidence interval: [5.41, 14.00], $d_{z}=1.737$, BH-FDR-corrected), MSCFormer by 4.50% (p=0.013, 95% confidence interval: [1.57, 7.43], $d_{z}=1.182$, BH-FDR-corrected), and CTNet by 3.15% (p=0.015, 95% confidence interval: [0.95, 5.35], $d_{z}=1.099$, BH-FDR-corrected). The gains over the competitive temporal-context modeling baselines ATCNet and TCFormer were relatively modest but remained statistically significant, with improvements of 1.77% (p=0.042, 95% confidence interval: [0.15, 3.40], $d_{z}=0.838$, BH-FDR-corrected) and 0.95% (p=0.043, 95% confidence interval: [0.04, 1.86], $d_{z}=0.802$, BH-FDR-corrected), respectively. Overall, these results confirm the competitive advantage of PG-MCTFormer in MI-EEG decoding, showing that the proposed prior-guided temporal–spatial modeling strategy improves classification performance consistently while maintaining strong robustness across subjects.

The subject-wise results reveal additional variation beyond the mean performance alone. PG-MCTFormer attains high accuracy on several subjects and remains competitive on difficult subjects such as S1, S2, S4, S6, S7 and S8, where the performance of other methods is lower [28,31,52].

**Figure 7:**
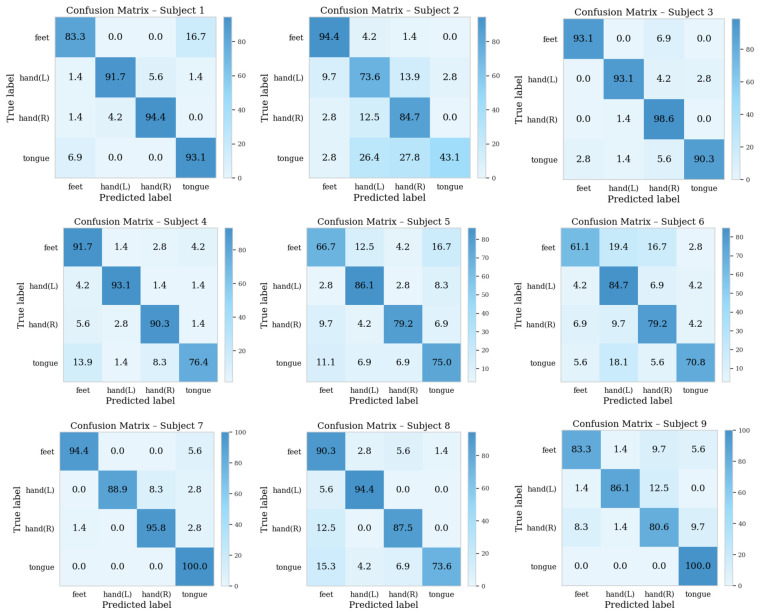
Confusion matrices of PG-MCTFormer across the nine BCIC IV-2a subjects.

Figure 7 presents the subject-wise confusion matrices of PG-MCTFormer. All four imagery classes are decodable across subjects, but the residual error pattern is clearly subject-dependent. For high-performing subjects such as S1, S3, S4, S7 and S9, the diagonal remains consistently strong, whereas the more difficult subjects S2, S5, S6, and S8 exhibit broader cross-class overlap. Hand-related confusions are still visible in several subjects, but notable tongue–hand overlap in S2, feet–tongue overlap in S1, S5, and S8, and the more diffuse mixing observed in S6 indicate that the dominant errors are not restricted to left- versus right-hand imagery alone.

## 5. Discussion

### 5.1. Ablation Studies

To systematically evaluate the contributions of the proposed components and their optimal configurations, we conducted ablation studies under the same session-wise protocol on BCIC IV-2a. The analysis was organized at two levels: a functional-module study that examined the contribution of the proposed temporal and spatial components, and hyperparameter studies that varied key temporal and spatial design choices while keeping all remaining settings fixed to the reported configuration.

#### 5.1.1. Ablation Study on Functional Modules

To assess the contribution of the proposed temporal and spatial priors and the S&R data augmentation strategy, ablation experiments were conducted on PG-MCTFormer while keeping the remaining settings unchanged. For the augmentation ablation, the full model architecture was retained and trained without S&R augmentation. For the structural ablations, the MS-STF was replaced with an unconstrained multi-kernel temporal convolution, the GDB-SC was replaced with the original depthwise spatial convolution, and both replacements were applied simultaneously. All ablation results were averaged over three independent random runs to reduce the influence of random initialization.

Table 2 compares five variants: the full model without S&R augmentation (w/o S&R Aug.), a prior-free decoder without both proposed priors (w/o Both Priors), a variant retaining only MS-STF (w/o GDB-SC), a variant retaining only GDB-SC (w/o MS-STF), and the full PG-MCTFormer. Removing S&R augmentation decreases the average accuracy from 85.08% to 83.32% (p=0.020, two-sided Wilcoxon signed-rank test), suggesting that data augmentation contributes to improving the robustness of the proposed model. For the structural priors, the prior-free decoder achieves an average accuracy of 84.13%. Retaining only MS-STF improves the accuracy to 84.45%, whereas retaining only GDB-SC yields a larger gain to 84.85%. Integrating both modules in the full model further raises the average accuracy to 85.08% with the best Cohen’s κ of 0.80. Although the gains are not uniform across all subjects, the overall results indicate that S&R augmentation, prior-guided temporal filtering, and grouped dual-branch spatial convolution provide complementary benefits for MI-EEG decoding.

#### 5.1.2. Ablation Study on Temporal Hyperparameters

To examine the sensitivity of the temporal frontend, we performed controlled ablations on four groups of hyperparameters within MS-STF: kernel lengths, the number of filters per branch, the combination of rhythm priors, and the maximum cutoff adaptation range. For each group, only the target setting was changed, while the remaining temporal and spatial configurations were fixed to the reported PG-MCTFormer setup. The results are summarized in Table 3.

Several observations can be drawn from Table 3. First, uniform kernel lengths are consistently suboptimal, whereas the asymmetric 65/33/21 allocation produces the best result, indicating that lower-frequency branches benefit from longer temporal support while higher-frequency branches benefit from shorter kernels. Second, a moderate capacity of 32 filters per branch provides the highest accuracy, while both smaller and larger configurations degrade performance on this small-sample benchmark. Third, incorporating the theta, mu, and beta priors together is more effective than using only beta or only mu+beta. Finally, bounded cutoff adaptation with 2 Hz yields the strongest result, suggesting that mild subject-specific spectral adjustment is beneficial, whereas excessive drift weakens the prior. The degradation observed after introducing the gamma branch is also consistent with the greater contamination of high-frequency scalp EEG by cranial muscle activity [53,54]. Overall, the temporal ablation supports a physiology-aligned interpretation of the MS-STF design rather than a purely capacity-driven one.

#### 5.1.3. Ablation Study on Spatial Hyperparameters

To examine the design choices within GDB-SC, we evaluated three groups of spatial ablations: the branch composition of the spatial module, the granularity of the motor-region prior, and the fusion strategy used before the grouped decoder. For the prior-granularity study, we compared four electrode grouping templates, namely Motor-4, Motor-6, Motor-8, and Motor-12; their detailed regional definitions are listed in Table 4.

As in the temporal study, only one factor was varied at a time, and all other settings were fixed to the reported configuration. The results are summarized in Table 5.

Table 5 shows that neither the fine branch nor the coarse branch alone reaches the performance of the dual-branch design, indicating that local electrode detail and region-level context are complementary. The granularity of the spatial prior also matters: the Motor-8 template performs best, whereas overly coarse (Motor-4) or overly fragmented (Motor-12) partitions reduce accuracy. The fusion arrangement further affects performance, as group-aligned interleaving clearly outperforms non-interleaved concatenation, suggesting the importance of preserving rhythm-specific grouping for the grouped decoder. Finally, to assess whether input-dependent adaptive fusion can further improve fine–coarse branch integration, we compared the proposed learnable per-group gate with a Squeeze-and-Excitation (SE)-based adaptive gate, in which the fine/coarse weights are dynamically generated from each input sample. The proposed gate achieves higher accuracy than the SE-based adaptive gate while reducing the parameter count from 166.2 K to 128.9 K, demonstrating its advantage in both model efficiency and decoding performance.

### 5.2. Interpretability Analyses

The prior-guided design of PG-MCTFormer enables the learned representation to be examined from complementary frequency, temporal, and spatial perspectives. Rather than relying on a single saliency view, the following analyses characterize the model at three different levels: frequency-domain visualization examines whether the learned Sinc filters remain aligned with physiologically meaningful bands; temporal visualization identifies the post-cue intervals that contribute most strongly to the final decision; and spatial visualization shows how the initialized sensorimotor prior is refined into subject-adaptive topographic patterns and how the model balances fine and coarse spatial pathways.

#### 5.2.1. Frequency-Domain Visualization

Frequency-domain analysis was performed by exporting the learned Sinc filters from each branch and computing their Fast Fourier Transform (FFT) magnitude responses. The spectra were peak-normalized and averaged first within subject and then across subjects. Following standard filter characterization, effective passbands were estimated using the −3 dB criterion, and the learned lower and upper cutoffs were summarized to quantify within-branch variability.

**Figure 8:**
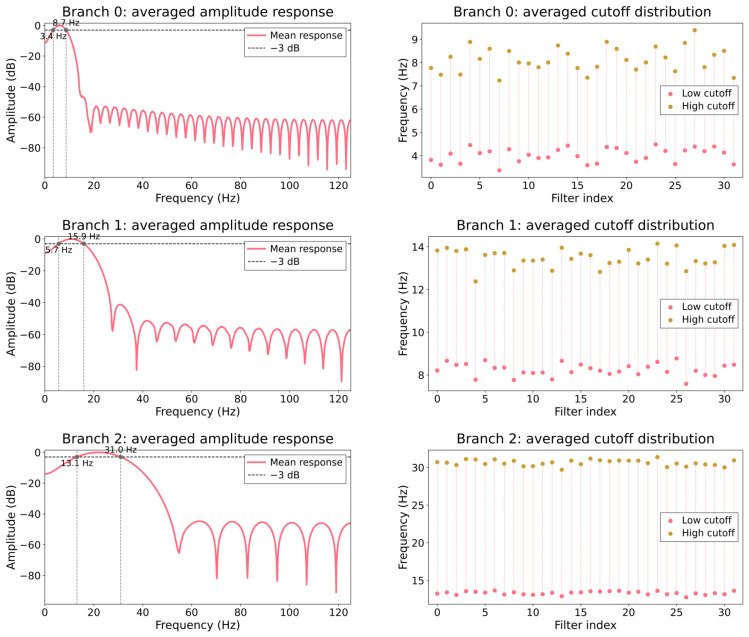
FFT responses and learned cutoff distributions of the three Sinc branches.

The MS-STF frontend provides direct access to the spectral characteristics learned by the three rhythm-specific branches. Figure 8 shows that the averaged FFT responses remain concentrated around the intended physiological ranges rather than drifting toward arbitrary bands. Using the −3 dB criterion, the effective passbands are approximately 3.4–8.7 Hz, 5.7–15.9 Hz, and 13.1–31.0 Hz for the three branches, respectively. The mu- and beta-oriented branches therefore remain closely aligned with the canonical sensorimotor rhythms most commonly implicated in MI-EEG classification [41,55,56], while the lower-frequency branch remains confined to a theta-related range rather than collapsing into the neighboring bands. The corresponding cutoff distributions further indicate that the filters do not collapse to a single identical response, but retain moderate within-branch diversity around the prescribed priors. These observations suggest that the temporal frontend preserves physiologically meaningful rhythm selectivity while still allowing limited subject-adaptive spectral refinement.

#### 5.2.2. Temporal Visualization

Gradient-weighted Class Activation Mapping (Grad-CAM) is a gradient-based visualization method that localizes the input regions contributing most strongly to a target decision [57]. In the original formulation, Grad-CAM is derived from the gradients flowing into the final convolutional feature maps, which preserve the ordered temporal or spatial layout and encode high-level representations closely related to the final classification decision. The resulting map therefore provides a coarse class-discriminative localization over learned feature bins. In the present study, one-dimensional Grad-CAM was computed from the second dilated causal convolutional layer in the last residual block of the grouped TCN head, with the class-specific pre-softmax logit used as the target score. The gradients were globally averaged over time to obtain channel weights,

(23) $$\alpha_{k}^{c}=\frac{1}{T^{\prime }}\sum_{t}\frac{\partial y^{c}}{\partial F_{k}\left(t\right)},$$

which were then used to construct the temporal saliency map

(24) $${\mathrm{CAM}}^{c}\left(t\right)=\mathrm{ReLU}\left(\sum_{k}\alpha_{k}^{c}F_{k}\left(t\right)\right).$$

where $F_{k}\left(t\right)$ denotes the *k*th feature map at pooled time bin *t* and $y^{c}$ denotes the pre-softmax logit for class *c*. Because the target layer lies after temporal pooling, the saliency is represented by 17 pooled time bins.

**Figure 9:**
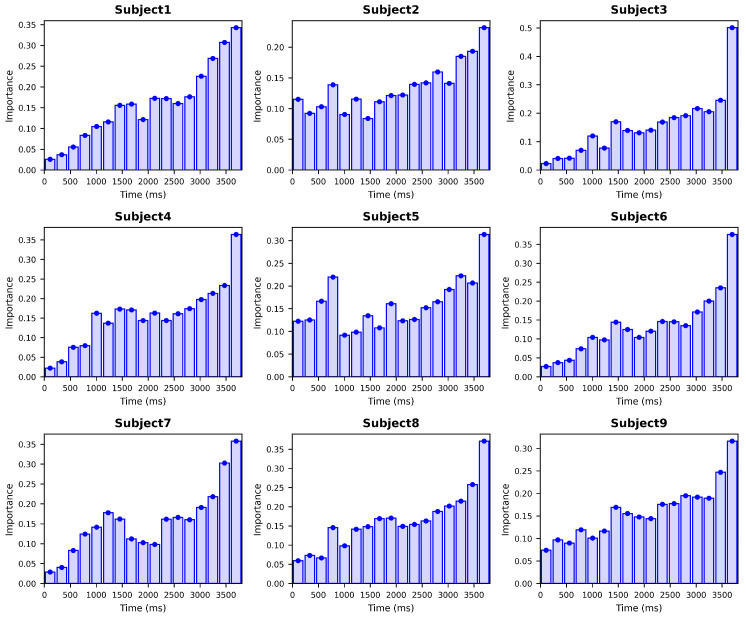
One-dimensional Grad-CAM curves from the final grouped TCN block.

Figure 9 presents the resulting subject-wise temporal importance curves. Across subjects, discriminative evidence generally accumulates from the middle portion of the imagery interval toward the later pooled bins, indicating that the model relies more strongly on sustained imagery-related activity than on the earliest post-cue response alone. This pattern is broadly consistent with prior MI studies showing that event-related desynchronization and related discriminative activity develop after cue onset and remain informative during sustained imagery [33,39,41]. At the same time, the curves exhibit clear inter-subject variation, which agrees with previous observations that the optimal temporal support of MI-EEG classification is subject-dependent rather than fixed across individuals [39]. Because the target layer is located after temporal pooling and the TCN head, the resulting Grad-CAM has the resolution of the pooled feature map and should therefore be interpreted as an overall temporal trend of decision-relevant evidence across the pooled bins [58,59].

#### 5.2.3. Spatial Visualization

To interpret the learned spatial organization, we examined two complementary quantities: the learned electrode-to-region assignment matrix W and the fine/coarse branch gate weights. Specifically, each row of W was projected onto a scalp topography to visualize how the initialized spatial prior evolved after training, whereas the three per-group gate values were read directly from the softmax-normalized gating logits to summarize the model-level balance between electrode-level and region-level processing.

**Figure 10:**
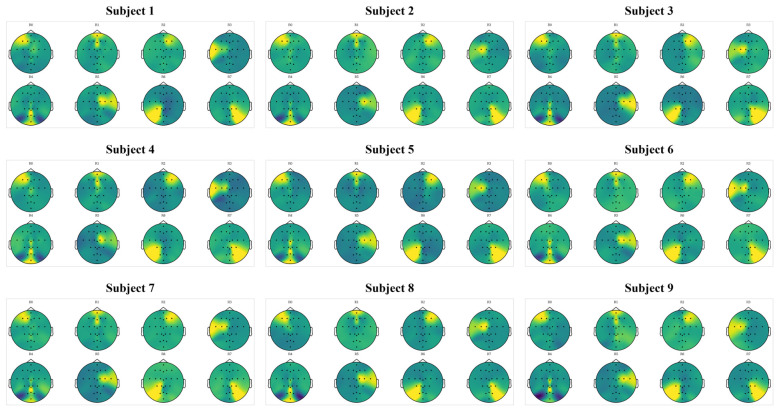
Learned rows of the soft assignment matrix W visualized as scalp topographies. Yellow regions indicate electrodes with higher learned assignment weights, suggesting greater contribution to the corresponding region-level feature aggregation, while black dots denote the EEG electrode positions.

Figure 10 visualizes the learned rows of the assignment matrix W. The resulting maps remain organized around a frontal-central, central, and centro-parietal sensorimotor axis rather than becoming spatially diffuse, suggesting that the initialized prior is retained in a data-adapted form. At the same time, the maps are not identical across regions, indicating that the model does not simply preserve the initialization but refines it into distinct spatial patterns. These topographies are broadly consistent with previous MI studies reporting both localized sensorimotor involvement and broader network-level participation during motor imagery [43,44,60]. In particular, the stronger weights near central electrodes are compatible with the well-established importance of sensorimotor areas in MI-EEG decoding [40,61].

From a connectivity-informed perspective, these topographies suggest that *W* defines neurophysiologically plausible regional nodes for coarse-scale interaction modeling. The learned projection aggregates electrode-level activity into region-level representations spanning fronto-central, central, and centro-parietal scalp areas, which is consistent with the distributed sensorimotor and fronto-parietal organization of motor imagery discussed above. Importantly, *W* provides a connectivity-informed sensor-to-region projection that bridges electrode-level EEG signals and region-level MI-related spatial organization [62].

**Figure 11:**
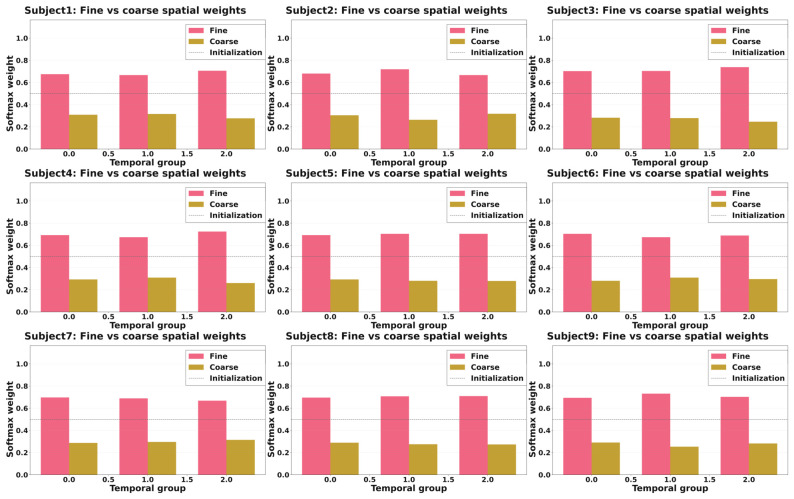
Fine/coarse branch gate weights for the three temporal groups.

Figure 11 further summarizes the branch preferences learned by the dual-pathway fusion mechanism. Across subjects and rhythm groups, the fine branch generally receives the larger weight, while the coarse branch also maintains a stable contribution. This pattern indicates that electrode-level detail is more strongly emphasized in the final representation, while region-level organization still provides complementary spatial context.

#### 5.2.4. Visualization with t-SNE

**Figure 12:**
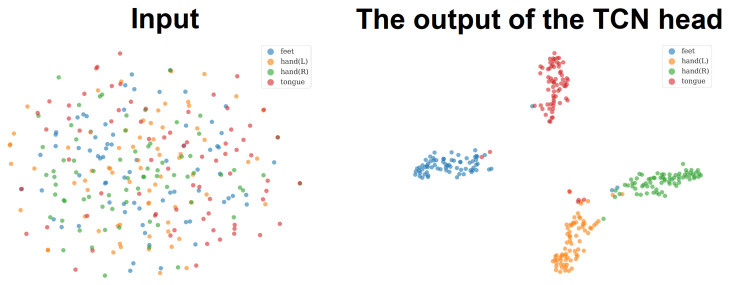
The t-SNE comparison between the raw input space and the pre-classifier representation for Subject 3. Each point corresponds to one test trial, and colors indicate the four MI classes.

The t-distributed Stochastic Neighbor Embedding (t-SNE) is a nonlinear dimensionality-reduction method that preserves local neighborhood structure when projecting high-dimensional samples into a low-dimensional space for visualization [63]. In this study, we applied t-SNE separately to the raw input space and to the output features of the TCN head immediately before the final classifier. Figure 12 shows the projections obtained from the same set of test trials from Subject 3 in the two spaces.

The raw input space exhibits substantial overlap among the four MI classes, with diffuse class boundaries and little visible cluster structure. In contrast, the learned representation at the output of the TCN head forms four markedly more compact and separated clusters. In particular, the feet, tongue, and right-hand classes become well isolated, while only limited boundary proximity remains between the two hand-related classes and a few scattered outliers. This qualitative comparison suggests that the proposed architecture transforms the raw EEG into a representation with clearer class organization before the final decision layer.

### 5.3. Computational Complexity and Inference Efficiency

For MI-BCI systems, single-trial decoding requires not only high classification accuracy but also acceptable computational cost. To profile the efficiency of PG-MCTFormer, we compared it with representative baselines in terms of trainable parameters, FLOPs, CPU inference latency, and GPU inference latency. All models were evaluated with the same input configuration on a workstation equipped with an NVIDIA GeForce RTX 5090 GPU and an x86_64 CPU. The number of parameters was computed from trainable weights. FLOPs were estimated by THOP for a single forward pass and reported as 2 × MACs. Inference latency was measured in evaluation mode over 2500 forward passes, and the mean latency was reported.

As detailed in Table 6, PG-MCTFormer contains 128.9 K trainable parameters, which is comparable to TCFormer (127.2 K), lower than MSCFormer (150.7 K) and CTNet (152.7 K), and much smaller than EEG Conformer (789.6 K). Although PG-MCTFormer is not the most lightweight model in terms of overall computational complexity, it achieves significantly higher average classification accuracy (85.08%) than the compared methods, indicating a favorable trade-off between computational cost and decoding performance. For inference latency, PG-MCTFormer achieves 11.41 ms on CPU, lower than TCFormer (13.27 ms), partly due to the eval-time caching of Sinc filters that reduces repeated filter construction. On GPU, PG-MCTFormer requires 4.29 ms per trial, which is far below the 4 s motor imagery interval of the BCI Competition IV 2a paradigm, supporting its practical feasibility for low-latency MI-BCI inference. Overall, these results indicate that PG-MCTFormer achieves the best average decoding accuracy with a moderate parameter scale and low inference latency.

## 6. Conclusions

In this study, we proposed PG-MCTFormer for MI-EEG classification by integrating rhythm-aware temporal filtering and topology-guided dual-scale spatial modeling into a modern convolutional-Transformer decoder. Specifically, the Multi-Scale Sinc Temporal Filterbank (MS-STF) constrains temporal filtering around the theta, mu, and beta priors, and the Gated Dual-Branch Spatial Clustering module (GDB-SC) combines electrode-level detail with region-level sensorimotor organization. Under the official session-wise protocol on BCI Competition IV 2a dataset, PG-MCTFormer achieved 85.08% average accuracy and a Cohen’s κ of 0.80, with statistically significant performance improvement over the compared MI-EEG decoding models. The ablation studies further confirmed that the temporal and spatial priors provide complementary gains. Together with the frequency-domain, temporal-domain, and spatial-domain analyses, these results indicate that explicit neurophysiological priors can improve not only classification performance but also the neurophysiological interpretability of modern MI-EEG decoders.

In our future work, we will evaluate the proposed framework on additional datasets and clinical MI scenarios, and further investigate transfer learning, calibration-light adaptation, and subject-level statistical associations between learned representations and classification performance.

## Figures and Tables

**Table 1:** Classification performance comparison on the BCI Competition IV 2a dataset. The results are presented in the format of Accuracy (%) and (Cohen’s κ). The best accuracy results for each subject and the average row are highlighted in bold. Avg. average, Std. standard deviation.

Sub.	EEGNet [25]	NexusNet [36]	EEG- Conformer [29]	MSCFormer [52]	CTNet [30]	ATCNet [28]	TCFormer [31]	PG- MCTFormer (Proposed)
S1	77.78 (0.70)	80.21 (0.74)	85.19 (0.80)	86.00 (0.81)	87.15 (0.83)	89.00 (0.85)	88.31 (0.84)	**89.93** (0.87)
S2	58.80 (0.45)	45.84 (0.28)	56.02 (0.41)	61.81 (0.49)	67.71 (0.57)	71.30 (0.62)	74.42 (0.66)	**75.12** (0.67)
S3	87.04 (0.83)	86.11 (0.81)	88.43 (0.85)	**94.68** (0.93)	92.48 (0.90)	94.33 (0.92)	**94.68** (0.93)	93.86 (0.92)
S4	66.32 (0.55)	61.46 (0.49)	75.12 (0.67)	81.25 (0.75)	82.06 (0.76)	80.32 (0.74)	84.61 (0.79)	**86.34** (0.82)
S5	62.85 (0.50)	70.02 (0.60)	59.84 (0.46)	73.15 (0.64)	**78.36** (0.71)	74.77 (0.66)	73.26 (0.64)	75.81 (0.68)
S6	58.68 (0.45)	53.70 (0.38)	59.95 (0.47)	70.02 (0.60)	68.40 (0.58)	72.46 (0.63)	72.46 (0.63)	**74.19** (0.66)
S7	84.26 (0.79)	71.41 (0.62)	90.74 (0.88)	89.01 (0.85)	90.28 (0.87)	92.82 (0.90)	93.52 (0.91)	**94.91** (0.93)
S8	79.28 (0.72)	74.54 (0.66)	80.67 (0.74)	85.18 (0.80)	85.76 (0.81)	86.23 (0.82)	87.38 (0.83)	**87.96** (0.84)
S9	80.44 (0.74)	72.57 (0.63)	82.41 (0.77)	84.14 (0.79)	85.18 (0.80)	**88.54** (0.85)	**88.54** (0.85)	87.62 (0.83)
Avg.	72.83 (0.64)	68.43 (0.58)	75.37 (0.67)	80.58 (0.74)	81.93 (0.76)	83.31 (0.78)	84.13 (0.79)	**85.08** (**0.80**)
Std.	±11.16 (0.15)	±12.71 (0.17)	±13.39 (0.18)	±10.32 (0.14)	±8.88 (0.12)	±8.83 (0.12)	±8.63 (0.12)	±8.04 (±0.11)

**Table 2:** Comparison of ablation results across different model variants on BCI Competition IV 2a. Results are reported as Accuracy (%) and (Cohen’s κ). The best accuracy results for each subject and the average row are highlighted in bold.

Subject	w/o S&R Aug. (Proposed)	w/o Both Priors (w/o MS-STF & GDB-SC)	w/o GDB-SC (MS-STF Retained)	w/o MS-STF (GDB-SC Retained)	Proposed Model (PG-MCTFormer)
S1	88.54 (0.85)	88.31 (0.84)	**90.63** (0.88)	89.93 (0.87)	89.93 (0.87)
S2	70.14 (0.60)	74.42 (0.66)	70.14 (0.60)	**75.12** (0.67)	**75.12** (0.67)
S3	94.10 (0.92)	**94.68** (0.93)	**94.68** (0.93)	93.17 (0.91)	93.86 (0.92)
S4	83.22 (0.78)	84.61 (0.79)	**87.04** (0.83)	86.34 (0.82)	86.34 (0.82)
S5	73.84 (0.65)	73.26 (0.64)	75.81 (0.68)	**76.27** (0.68)	75.81 (0.68)
S6	74.54 (0.66)	72.46 (0.63)	**74.88** (0.67)	73.15 (0.64)	74.19 (0.66)
S7	92.36 (0.90)	93.52 (0.91)	92.01 (0.89)	93.29 (0.91)	**94.91** (0.93)
S8	86.34 (0.82)	87.38 (0.83)	86.11 (0.81)	**88.31** (0.84)	87.96 (0.84)
S9	86.80 (0.82)	88.54 (0.85)	**88.77** (0.85)	88.08 (0.84)	87.62 (0.83)
Avg.	83.32 (0.78)	84.13 (0.79)	84.45 (0.79)	84.85 (0.80)	**85.08** (**0.80**)
Std.	±8.57 (±0.11)	±8.63 (±0.12)	±8.65 (±0.12)	±7.87 (±0.10)	±8.04 (±0.11)

**Table 3:** Average accuracy of temporal-design ablations on BCI Competition IV 2a. Bold values indicate the best-performing setting and the corresponding accuracy within each ablation category.

Category	Setting	Accuracy (%)
Kernel lengths	21/21/21 for θ, μ, and β	83.02
	33/33/33 for θ, μ, and β	83.53
	65/65/65 for θ, μ, and β	81.10
	**65/33/21 for θ, μ, and β**	**85.07**
Filters per branch	16	83.49
	**32**	**85.07**
	64	84.65
	128	83.10
Band priors	β [13–30 Hz]	73.15
	μ [8–13 Hz] + β [13–30 Hz]	83.49
	**θ [4–8 Hz] + μ [8–13 Hz] + β [13–30 Hz]**	**85.07**
	θ [4–8 Hz] + μ [8–13 Hz] + β [13–30 Hz] + γ [30–40 Hz]	82.99
Boundary adaptation	0 Hz	83.26
	1 Hz	83.60
	**2 Hz**	**85.07**
	4 Hz	84.15

**Table 4:** Definitions of the motor-region grouping templates used in the spatial-prior ablations.

Template	Regional Grouping					
Motor-4	**R1**: {FC3, FC1, C5, C3, C1, CP3, CP1, P1}			**R2**: {Fz, FCz, Cz}		
	**R3**: {FC2, FC4, C2, C4, C6, CP2, CP4, P2}			**R4**: {CPz, Pz, POz}		
Motor-6	**R1**: {FC3, FC1, C5, C3, C1}			**R2**: {Fz, FCz, Cz}		
	**R3**: {FC2, FC4, C2, C4, C6}			**R4**: {CP3, CP1, P1}		
	**R5**: {CPz, Pz, POz}			**R6**: {CP2, CP4, P2}		
Motor-8	**R1**: {FC3, FC1}			**R2**: {Fz, FCz}		
	**R3**: {FC2, FC4}			**R4**: {C5, C3, C1}		
	**R5**: {Cz, CPz, Pz, POz}			**R6**: {C2, C4, C6}		
	**R7**: {CP3, CP1, P1}			**R8**: {CP2, CP4, P2}		
Motor-12	**R1**: {FC3, FC1}		**R2**: {Fz, FCz}		**R3**: {FC2, FC4}	
	**R4**: {C5, C3, C1}		**R5**: {Cz}		**R6**: {C2, C4, C6}	
	**R7**: {CP3, CP1}		**R8**: {CPz}		**R9**: {CP2, CP4}	
	**R10**: {P1}		**R11**: {Pz, POz}		**R12**: {P2}	

**Table 5:** Average accuracy of spatial-design ablations on BCI Competition IV 2a. Bold values indicate the best-performing setting and the corresponding accuracy within each ablation category.

Category	Setting	Accuracy (%)
Spatial branch	Fine only (channel-wise depthwise)	84.49
	Coarse only (region-level projection)	83.99
	**Dual-branch fusion**	**85.07**
Spatial prior	Without prior	83.85
	Motor-4	83.56
	Motor-6	84.34
	**Motor-8**	**85.07**
	Motor-12	83.22
Fusion mode	Non-interleaved concatenation	82.79
	**Group-aligned interleaving**	**85.07**
Gating strategy	SE-based adaptive gate	84.68
	**Learnable per-group gate**	**85.07**

**Table 6:** Computational complexity and single-trial inference efficiency on the BCI Competition IV 2a dataset.

Model	Params (K)	FLOPs (M)	CPU Infer. Time (ms)	GPU Infer. Time (ms)
EEGNet [25]	1.7	3.37	0.35 ± 0.020	0.20 ± 0.006
NexusNet [36]	3.3	1.56	1.28 ± 0.017	0.55 ± 0.001
EEG Conformer [29]	789.6	131.29	4.44 ± 0.082	1.90 ± 0.013
MSCFormer [52]	150.7	145.77	3.26 ± 0.089	1.89 ± 0.009
CTNet [30]	152.7	71.89	2.78 ± 0.068	1.83 ± 0.009
ATCNet [28]	113.7	52.77	4.11 ± 0.120	5.39 ± 0.023
TCFormer [31]	127.2	188.71	13.27 ± 0.312	4.02 ± 0.008
PG-MCTFormer (Proposed)	128.9	211.29	11.41 ± 0.204	4.29 ± 0.006

## Data Availability

A publicly available dataset was analyzed in this study. This data can be found here: BCI Competition IV 2a dataset http://www.bbci.de/competition/iv/ (accessed on 12 December 2025).
